# Falling bacterial communities from the atmosphere

**DOI:** 10.1186/s40793-020-00369-4

**Published:** 2020-12-10

**Authors:** Cheolwoon Woo, Naomichi Yamamoto

**Affiliations:** 1 Department of Environmental Health Sciences, Graduate School of Public Health, Seoul National University, Seoul, 08826 Republic of Korea; 2 Institute of Health and Environment, Seoul National University, Seoul, 08826 Republic of Korea

**Keywords:** Bioprecipitation, Biosedimentation, Bioaerosols, 16S rRNA gene, Aerobiology, Aero-microbiology

## Abstract

**Background:**

Bacteria emitted into the atmosphere eventually settle to the pedosphere via sedimentation (dry deposition) or precipitation (wet deposition), constituting a part of the global cycling of substances on Earth, including the water cycle. In this study, we aim to investigate the taxonomic compositions and flux densities of bacterial deposition, for which little is known regarding the relative contributions of each mode of atmospheric deposition, the taxonomic structures and memberships, and the aerodynamic properties in the atmosphere.

**Results:**

Precipitation was found to dominate atmospheric bacterial deposition, contributing to 95% of the total flux density at our sampling site in Korea, while bacterial communities in precipitation were significantly different from those in sedimentation, in terms of both their structures and memberships. Large aerodynamic diameters of atmospheric bacteria were observed, with an annual mean of 8.84 μm, which appears to be related to their large sedimentation velocities, with an annual mean of 1.72 cm s^− 1^ for all bacterial taxa combined. The observed mean sedimentation velocity for atmospheric bacteria was larger than the previously reported mean sedimentation velocities for fungi and plants.

**Conclusions:**

Large aerodynamic diameters of atmospheric bacteria, which are likely due to the aggregation and/or attachment to other larger particles, are thought to contribute to large sedimentation velocities, high efficiencies as cloud nuclei, and large amounts of precipitation of atmospheric bacteria. Moreover, the different microbiotas between precipitation and sedimentation might indicate specific bacterial involvement and/or selective bacterial growth in clouds. Overall, our findings add novel insight into how bacteria participate in atmospheric processes and material circulations, including hydrological circulation, on Earth.

**Supplementary Information:**

The online version contains supplementary material available at 10.1186/s40793-020-00369-4.

## Background

Bacteria are ubiquitous in the lithosphere [1], hydrosphere [2], and atmosphere [3, 4]. Although the number of bacterial species is still not accurately known, a study [5] reported 0.8–1.6 million operational taxonomic units (OTUs) based on sequences of the V4 region of the 16S rRNA gene at a 97% sequence similarity threshold. Bacteria are emitted into the atmosphere with estimated global rates of 0.7–28.1 Tg y^− 1^ [6]. The emitted bacteria play roles in ecological and climate systems, for example, by acting as cloud condensation nuclei and/or ice-forming nuclei [6–8]. Moreover, the emitted bacteria eventually settle to the pedosphere by sedimentation (dry deposition) or precipitation (wet deposition), constituting part of the global cycling of substances on Earth, including the water cycle [9].

Bacterial cells are small in size (0.3–10 μm) compared to fungal spores (0.5–30 μm) and pollen grains (10–100 μm) [10, 11]. Small bacterial cells can create small sedimentation velocities, long residence times in the atmosphere, and, therefore, long-range transport, for example, from Asia to North America [12, 13]. Meanwhile, bacteria can also aggregate and/or attach to other particles to form larger particles [14–16]. Large particles not only generate large sedimentation velocities and flux densities [17–19] but also promote precipitation by serving as giant cloud condensation nuclei, since giant particles (> 2 μm), and, in particular, ultra-giant particles (> 10 μm), can effectively collide and scavenge smaller droplets in the air [20, 21]. A recent study [16] reported that precipitation dominated bacterial but not viral deposition, indicating a possibility that larger bacterial particles might selectively serve as giant cloud condensation nuclei.

In addition to particle size, particles’ physicochemical properties can also affect nucleation activities. For instance, *Pseudomonas syringae* is a well-known bacterial species that produces ice nucleation proteins [22–24] and promotes nucleation in the atmosphere. Similarly, several ice-nucleation active bacteria have been reported [25–28] and isolated from clouds [29, 30] and precipitation, including rainfall [9] and snowfall [9, 31, 32]. Thus, bacteria likely play a role and are involved in precipitation, which is generally referred to as “bioprecipitation” [33, 34]. However, it is still controversial to what extent biological particles, including bacterial particles, contribute to nucleation and are involved in precipitation on a global scale [7, 35–37]. In addition, little is known regarding how precipitation is related to bacterial communities in the atmosphere.

The goal of this study is to investigate the taxonomic compositions and flux densities of bacterial precipitation and sedimentation from the atmosphere. To date, several studies have reported bacterial communities in precipitation [31, 36, 38–41]. However, few studies exist to compare bacterial communities in precipitation to those in sedimentation [39]. The comparison is needed to identify taxa that are uniquely related to precipitation. Moreover, knowledge is scarce regarding taxon-specific bacterial particle sizes [42] that can influence sedimentation velocities and nucleation efficiencies. In our previous fungal study [17], we found that mushroom-forming agaricomycetes were enriched and specifically involved in precipitation. Here, we wished to extend our investigation into bacterial analyses. Specifically, we reanalyze air, sedimentation, and precipitation samples that were previously collected in Seoul in South Korea from May to November 2015 for our fungal [17] and plant [43] analyses. These samples were collected by an automatic dry and wet deposition sampler [44] and a collocated eight-stage Andersen sampler [45]. The samples are reanalyzed here by a bacterial-specific quantitative PCR (qPCR) assay and Illumina MiSeq sequencing, targeting the V3 and V4 regions of bacterial 16S rRNA gene.

## Results

### Sample statistics

In total, 30 air samples, collected monthly from May to November 2015, each month with five particle size bins, were sequenced for V3 and V4 regions of the bacterial 16S rRNA gene (Table S1). Air sampling for August failed due to an intense precipitation event. Deposition samples were collected monthly in duplicates from May to November 2015, resulting in a total of 28 libraries consisting of 14 precipitation (wet deposition) and 14 sedimentation (dry deposition) libraries. In total, 1,262,866 and 1,119,431 high-quality sequence reads were obtained from 30 air sample libraries and 28 deposition libraries, respectively (Table S1). The number of sequence reads ranged from 8425 to 61,239 reads per library. The sequencing depth seems adequate for α-diversity analyses, since the rarefaction curves appear to reach to asymptotes, except for two libraries for air samples collected in May (Fig. S1).

### Deposition

**Fig. 1:**
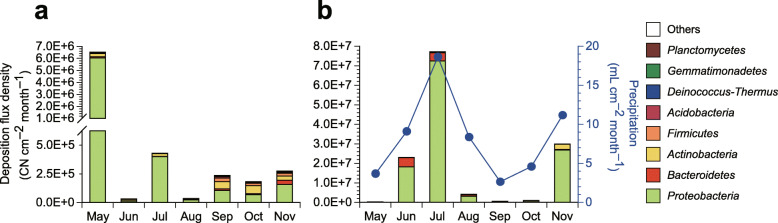
Bacterial deposition from the atmosphere. **a** Flux densities of bacterial deposition in the dry form. **b** Flux densities of bacterial deposition in the wet form. The precipitation data [17] are shown on the secondary axis

Flux densities of atmospheric bacterial depositions were seasonally varied, with peaks observed in May for sedimentation and in July for precipitation (Fig. 1a,b). Similar seasonal trends and strong correlation were observed between the flux densities of bacterial wet deposition and the amounts of precipitation, with their peaks observed in July (Fig. 1b) and with Spearman’s *ρ* = 0.96 (Fig. S2). The annually averaged flux densities were 1,100,000 and 19,400,000 copy number (CN) of 16S rRNA gene cm^− 2^ month^− 1^ (CN cm^− 2^ month^− 1^) for dry and wet deposition, respectively, indicating that 95 and 5% of bacteria were precipitated and sedimented, respectively, from the atmosphere. However, the month-based contributions of wet deposition seasonally varied from 4.1% in May to 99.9% in June (Table S2).

**Fig. 2:**
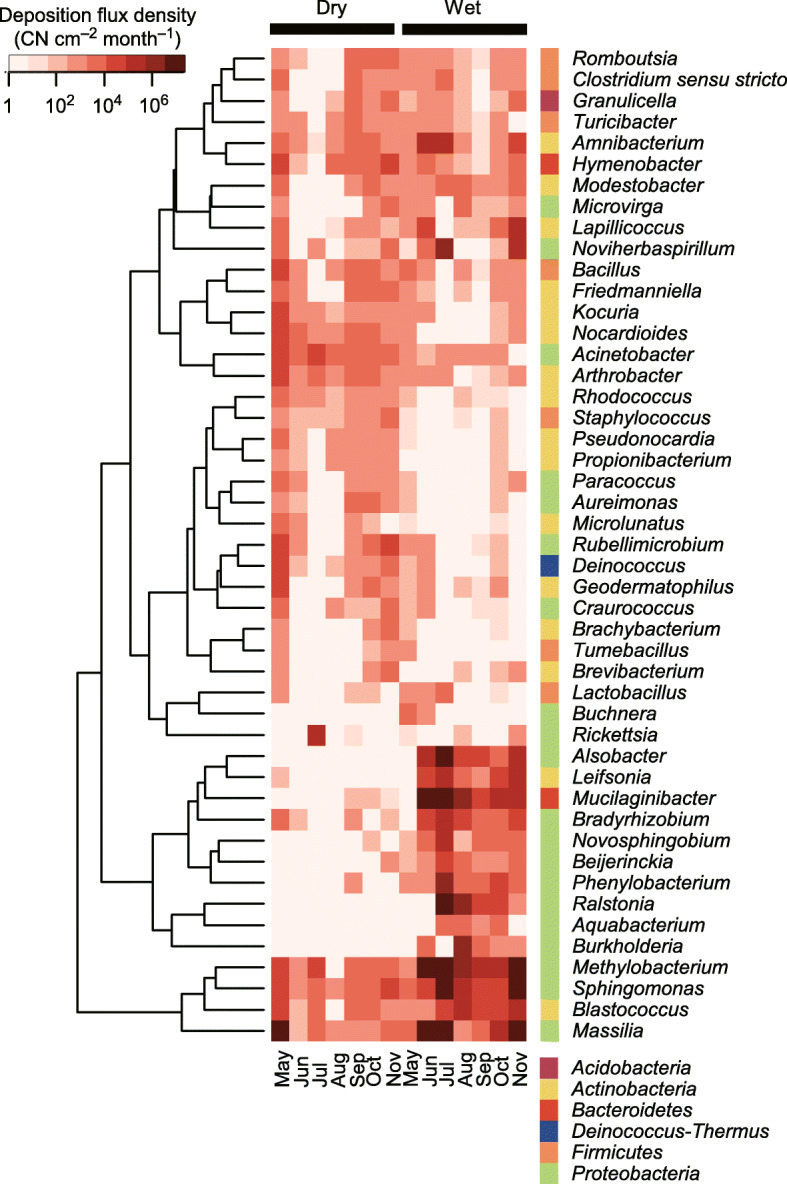
Flux densities of atmospheric deposition of selected bacterial genera. The 30 most abundant genera in dry and/or wet deposition in terms of the sequence relative abundances are shown. The tree represents the similarities of the log-transformed abundances across genera based on Euclidean distances

The three most abundant phyla and their mean flux densities were *Proteobacteria* (952,000 CN cm^− 2^ month^− 1^), *Actinobacteria* (63,000 CN cm^− 2^ month^− 1^), and *Bacteroidetes* (19,000 CN cm^− 2^ month^− 1^) for dry deposition (Fig. 1a), and *Proteobacteria* (17,430,000 CN cm^− 2^ month^− 1^), *Bacteroidetes* (1,460,000 CN cm^− 2^ month^− 1^), and *Actinobacteria* (52,000 CN cm^− 2^ month^− 1^) for wet deposition (Fig. 1b). At the genus level, bacteria abundant in both dry and wet deposition included *Methylobacterium*, *Sphingomonas*, *Blastococcus*, and *Massilia* (Fig. 2). The genera specifically enriched in wet deposition included *Novosphingobium*, *Beijerinckia*, *Phenylobacterium*, *Ralstonia*, *Aquabacterium*, and *Burkholderia* (Fig. 2). *Pseudomonas* was detected at small relative contributions, i.e., 0.065 and 0.003% of total bacterial sedimentation and precipitation, respectively.

### Air

**Fig. 3:**
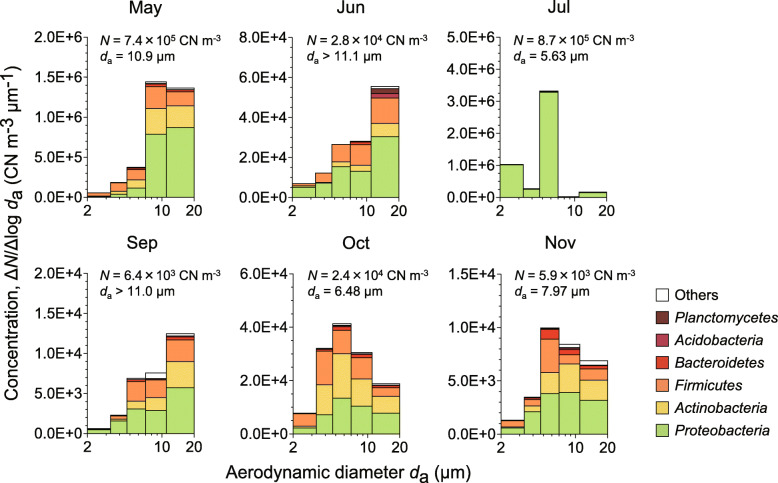
Particle size distributions of bacteria in the atmosphere. Particle-size integrated concentrations (*N*) are shown with the geometric means of the aerodynamic diameters (*d*_a_) of all bacterial taxa combined

The particle-size-integrated total bacterial concentrations seasonally varied from 5900 CN m^− 3^ to 870,000 CN m^− 3^ (Fig. 3), with their annual mean concentration of 280,000 CN m^− 3^. The geometric means of the aerodynamic diameters (*d*_a_) of bacterial particle size distribution range from 5.63 μm to > 11.1 μm (Fig. 3), with their annual mean of 8.84 μm, for all bacterial taxa combined. On an annual basis, 25% of bacteria were of a particle size of *d*_a_ > 11 μm, while the remaining 62 and 13% of bacteria were in the particle size range of *d*_a_ = 3.3–11 μm and *d*_a_ = 2.1–3.3 μm, respectively.

**Fig. 4:**
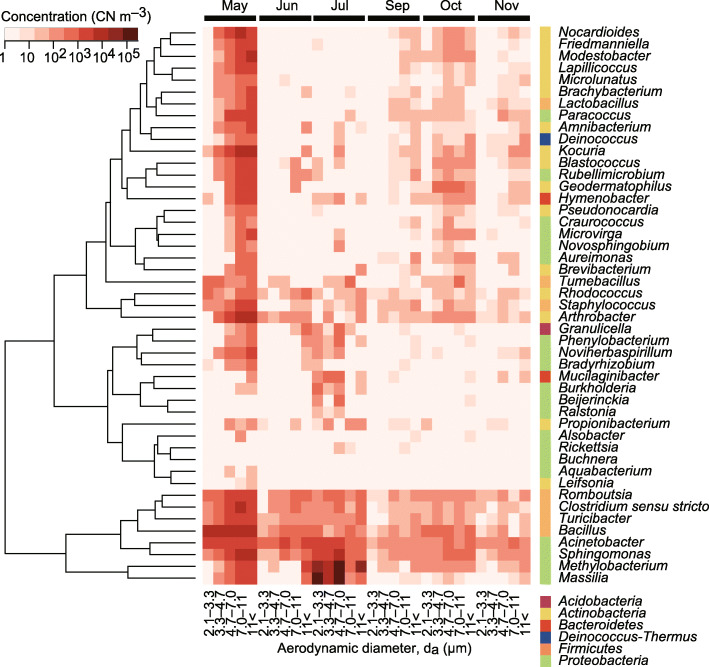
Particle size-resolved concentrations of selected bacterial genera. The genera in Fig. 2 are shown. The tree represents the similarities of the log-transformed abundances across genera based on Euclidean distances

The six most abundant phyla and their annual particle-size-integrated concentrations and aerodynamic diameters were *Proteobacteria* (214,000 CN m^− 3^, 9.02 μm), *Actinobacteria* (29,000 CN m^− 3^, 10.1 μm), *Firmicutes* (29,000 CN m^− 3^, 7.55 μm), *Bacteroidetes* (3200 CN m^− 3^, 8.85 μm), *Acidobacteria* (1100 CN m^− 3^, 8.91 μm), and *Planctomycetes* (570 CN m^− 3^, 10.4 μm) (Fig. 3). At the genus level (Fig. 4), the five most abundant bacteria and their annually averaged particle-size-integrated concentrations were *Methylobacterium* (66,000 CN m^− 3^), *Massilia* (62,000 CN m^− 3^), *Bacillus* (9300 CN m^− 3^), *Sphingomonas* (6700 CN m^− 3^), and *Acinetobacter* (4400 CN m^− 3^). The aerodynamic diameters of selected bacterial genera are shown in Table 1.

**Table 1:** Atmospheric properties of selected bacterial genera

Phylum	Genus ^a^	Sedimentation velocity, *V*_d_ (cm s^− 1^) ^b^	Aerodynamic diameter, *d*_a_ (μm) ^c^	Microscopy-based diameter or width × length [ref.]
*Actinobacteria*	*Arthrobacter*	0.64	8.34	0.6–1.0 μm [46]
	*Amnibacterium*	2.5	9.99	0.15–0.20 × 0.25–0.30 μm for *Amnibacterium kyonggiense* [47]
	*Blastococcus*	1.7	10.1	0.3–1.7 μm [48]
	*Rhodococcus*	1.5	8.74	0.4–0.6 × 8–12 μm for filaments and 0.2–0.5 × 1.7–3 μm for cocci or short rods for *Rhodococcus jostii* [49]
	*Kocuria*	0.42	9.31	1–1.5 μm for *Kocuria rosea* [50]
	*Brachybacterium*	0.54	10.3	0.5–0.75 × 1.5–2.5 μm for *Brachybacterium faecium* [51]
	*Propionibacterium*	1.6	8.54	0.2–1.5 × 1–5 μm for pleomorphic rods, 20 μm in length for filaments, and 5–20 μm for swollen spherical cells [52]
*Bacteroidetes*	*Mucilaginibacter*	0.086	5.32	0.43–0.49 × 1.4–1.8 μm for *Mucilaginibacter gynuensis* [53]
*Firmicutes*	*Bacillus*	0.18	6.75	0.4–1.8 × 0.9–10.0 μm [54]
	*Turicibacter*	0.30	8.15	0.5–2.0 × 0.7–7.0 μm [55]
	*Staphylococcus*	0.34	8.75	0.5–1.5 μm [56]
	*Romboutsia*	0.26	7.33	1.2–2 × 2.3–10 μm for *Romboutsia sedimentorum* [57]
	*Clostridium*	0.22	8.03	0.5–1.7 × 2.4–7.6 μm for *Clostridium butyricum* [58]
*Proteobacteria*	*Acinetobacter*	0.90	5.92	0.9–1.6 × 1.5–2.5 μm [59]
	*Massilia*	5.7	8.79	1.0 × 3.0 μm [60]
	*Sphingomonas*	0.92	9.90	0.2–1.4 × 0.5–4.0 μm [61]
	*Methylobacterium*	0.079	10.7	0.8–1.2 × 1.0–8.0 μm [62]
	*Rubellimicrobium*	2.1	10.9	0.6–0.8 × 2.0–4.0 μm for *Rubellimicrobium thermophilum* [63]
	*Paracoccus*	0.54	8.39	0.5–0.9 μm [64]
	*Noviherbaspirillum*	1.7	10.2	1.0–1.3 × 1.5–2.0 μm for *Noviherbaspirillum agri* [65]

^a^Genera shown in Fig. 2 and detected in air samples for all sampling months have been selected here.

^b^Calculated according to Eq. (1) in the method section. The data of August are excluded for the calculation.

^c^For each sampling month, a geometric mean of the aerodynamic diameters of a given particle size distribution was computed using the GM calculator version 1.0 [66]. Then, the mean of the computed monthly-based geometric means of the aerodynamic diameters was calculated to obtain an annually averaged aerodynamic diameter that is representative for each genus

### Diversity

**Fig. 5:**
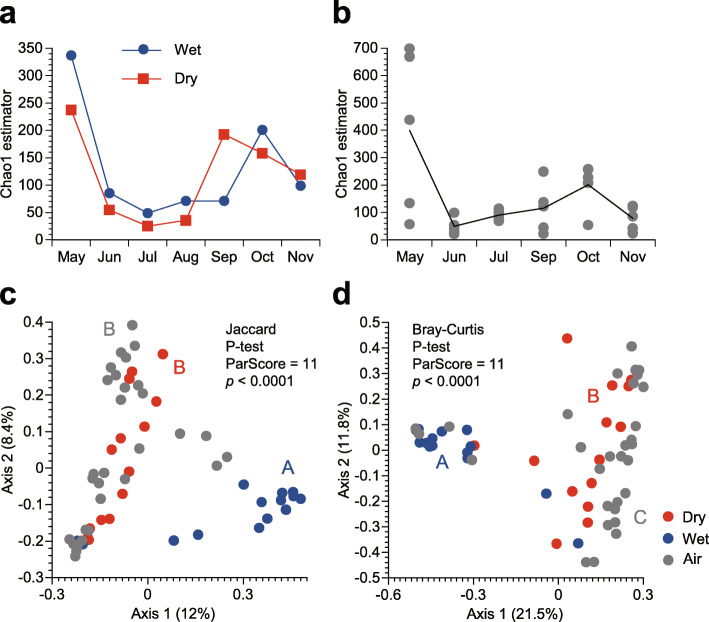
Bacterial diversity in deposition and air samples. **a** Chao1 estimates of the number of 97% of the operational taxonomic units (OTUs) in the deposition samples. **b** Chao1 estimates of the number of 97% of the OTUs in the air samples. Each point represents a datum of each particle size bin for each month. The line indicates the mean particle size-resolved results for each month. **c** Principal coordinate analysis plot of bacterial memberships in terms of the Jaccard indices, based on 97% OTUs. Significant differences for pair-wise comparisons are indicated by different letters (**a and b**). **d** Principal coordinate analysis plot of the bacterial structures in terms of the Bray–Curtis distances, based on 97% OTUs. Significant differences for pair-wise comparisons are indicated by different letters (**a, b, and c**)

Bacterial taxonomic richness was seasonally varied, with primary peaks observed in May for both dry and wet deposition, and secondary peaks in September for dry deposition and in October for wet deposition (Fig. 5a). These tendencies appear to reflect the seasonal tendency in taxonomic richness in the atmosphere, with the primary peak in May and secondary peaks in October (Fig. 5b). However, the future interannual observation is needed to confirm the reproducibility in the observed seasonal tendencies. Bacterial communities were statistically significantly different in terms of dry and wet deposition, both in terms of their memberships (*p* < 0.0001; P-test) (Fig. 5c) and structures (*p* < 0.0001; P-test) (Fig. 5d). No significant difference was observed in the bacterial memberships between dry deposition and the air (*p* > 0.05; P-test) (Fig. 5c).

### Sedimentation velocity

**Fig. 6:**
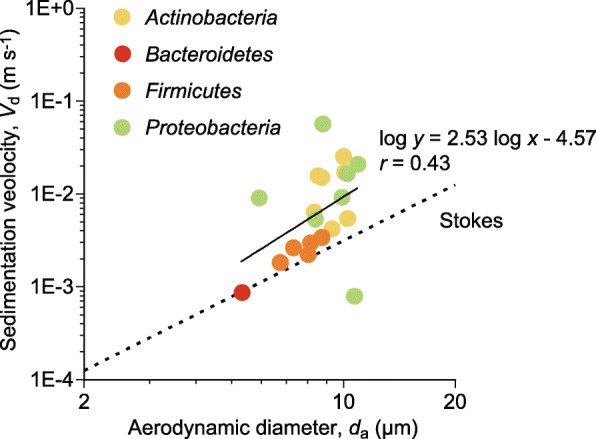
Relationship between the sedimentation velocities (*V*_d_) and aerodynamic diameters (*d*_a_) of atmospheric bacteria. Each point represents one datum for each genus. The genera in Table 1 are shown. Theoretical terminal settling velocities (*V*_Stk_), given by Stokes’ law (denoted by the dashed line), are included for comparison

Dry deposition (sedimentation) velocities (*V*_d_) were calculated according to Eq. (1), which is described in the method section. Briefly, the velocities were calculated by dividing the sedimentation flux densities, measured using the dry deposition sampler by air volumetric concentrations measured using the collocated Andersen sampler. The sedimentation velocity for all bacterial taxa combined was 1.72 cm s^− 1^. At the phylum level, the velocities were 2.00 cm s^− 1^ for *Proteobacteria*, 2.64 cm s^− 1^ for *Bacteroidetes*, 0.97 cm s^− 1^ for *Actinobacteria*, 0.26 cm s^− 1^ for *Firmicutes*, 1.23 cm s^− 1^ for *Acidobacteria*, and 0.23 cm s^− 1^ for *Planctomycetes*. The sedimentation velocities for the selected genera and their aerodynamic diameters are shown in Fig. 6 and are listed with their microscopy-based sizes in Table 1. A moderate positive correlation was observed between the sedimentation velocities and aerodynamic diameters for those selected genera (Pearson’s *r* = 0.43) (Fig. 6).

## Discussion

In this study, we measured the flux densities and characterized the taxonomic assemblages of bacteria deposited from the atmosphere in both dry (sedimentation) and wet (precipitation) modes. We also quantified bacterial aerodynamic diameters and sedimentation velocities by the collocation of a volumetric eight-stage Andersen sampler and an automated dry deposition sampler. We found that precipitation contributed to 95% of atmospheric bacterial deposition (Table 2) and that bacterial assemblages in precipitation were significantly different from those in sedimentation (*p* < 0.0001; P-test) (Fig. 5c,d). Moreover, we observed large aerodynamic diameters of atmospheric bacteria (Fig. 3), with an annual mean of 8.84 μm (Table 2), which might be related to their large sedimentation velocities (Fig. 6), with an annual mean of 1.72 cm s^− 1^ for all bacterial taxa combined (Table 2).

**Table 2:** Comparison of atmospheric properties of biological particles ^a^

Type	Contribution of precipitation to total deposition	Aerodynamic diameter, *d*_a_ (μm)	Sedimentation velocity, *V*_d_ (cm s^− 1^)	Reference
Bacteria	95%	8.84	1.72	This study
Fungi	86%	5.93	0.80	[17]
Plants	13%	n.d.	0.40	[43]

^a^The annually averaged values for all taxa combined are shown

*Abbreviation*: *n.d.* not determined due to skewed particle size distributions

### Large aerodynamic diameters of atmospheric bacteria

We found that the mean bacterial aerodynamic diameter (*d*_a_ = 8.84 μm) was larger than the previously characterized mean fungal aerodynamic diameter (*d*_a_ = 5.93 μm) [17], which is contrary to the reported microscopy-based sizes of 0.3–10 μm for bacterial cells and 0.5–30 μm for fungal spores [10, 11]. We also found that the bacterial genus-specific aerodynamic diameters measured in this study were consistently larger than their reported microscopy-based sizes (Table 1), indicating that bacteria aggregate and/or attach to other particles to form larger particles in the atmosphere. Indeed, existing studies [14–16] have reported bacterial aggregation and attachment to other particles.

The large observed aerodynamic diameters of atmospheric bacteria may be partially explained by the differences in how microbial quantities are characterized by our molecular biology-based approach. Traditionally, a culture-based approach has been used to characterize airborne microbial particle size distributions by enumerating microbial colonies grown on nutrient agar plates loaded in a multi-stage impactor, including the Andersen sampler [45, 67, 68]. For instance, the count median diameters of 2.4–4.8 μm and 3.1–4.5 μm were reported for airborne culturable bacteria by culture-based studies [69, 70]. However, this approach distorts microbial particle size distributions based on their biomass since it quantitates the number of colonies, each possibly derived from an aggregate of multiple microbial cells. For instance, it quantitates a smaller particle containing a single microbial cell and a larger particle containing multiple cells equivalently to 1 colony forming unit (CFU). This underestimates the microbial biomass in larger particles relative to that in smaller particles, resulting in a measured peak size that is smaller than the actual peak of a given microbial particle size distribution. In this study, we have quantified the number of bacterial 16S rRNA gene copies by qPCR, and this approach provides a more proportional measure of bacterial biomass. The proportionality might be possibly distorted by the variability in the number of 16S rRNA gene copies per cell [71]. However, there is no evidence that this variability is dependent on bacterial size, so we can reasonably assume that the qPCR-derived bacterial particle size distributions are likely to be proportional to those based on the bacterial biomass.

We observed that a small fraction (13%) of bacterial 16S rRNA gene copies were in a particle size range of *d*_a_ = 2.1–3.3 μm (Fig. 3). Due to the small bacterial cell size (0.3–10 μm) [10, 11], it is possible that they exist in smaller particle size fractions, which were not investigated in this study. Indeed, the literature has reported the detection of bacteria from fine particles where *d*_a_ < 2.5 μm (PM_2.5_) [72, 73]. Although they constitute relatively minor fractions, these small bacterial particles might contribute to the reported long-range transport (e.g., [12, 13]) due to long residence times and small sedimentation velocities in the atmosphere. Meanwhile, a larger fraction (25%) of bacteria was detected from a particle size fraction of *d*_a_ > 11 μm. Since ultra-giant particles (> 10 μm) are known to serve as effective giant cloud condensation nuclei [20], these bacterial particles might serve as giant cloud condensation nuclei [21]. Previous studies have reported that ultra-giant particles can reach cloud base altitudes, e.g., > 1000 m [74, 75], and that bacteria have been detected at cloud base altitudes (500–2000 m) [76] and even at higher altitudes of the stratosphere [77, 78].

We would also like to note large variability in peak aerodynamic diameters of atmospheric bacteria observed each month, ranging from *d*_a_ = 5.63 μm to *d*_a_ > 11.1 μm (Fig. 3). We found that the size peaks were inversely associated with the minimum and mean relative humidity observed during each sampling month, with Spearman’s *ρ* = − 0.77 and − 0.60, respectively, although the results are not statistically significant (Table S3). Particle resuspension (aerosolization) is known to be reduced with increased relative humidity [79]. One possible explanation is that the elevated relative humidity reduced aeolian aerosolization of local bacterial particles, including large-size bacterial particles, while small-size particles remained permitted from non-local distant sources due to their ability of long-range transport. We expect that these particle size-dependent imbalances might be a cause of selective reduction of large-size particles and therefore shifts in the size distributions observed during wet months (e.g., July and November) at our sampling site.

### Large sedimentation velocities of atmospheric bacteria

Large sedimentation velocities were observed for bacteria (Fig. 6 and Table 1), with a mean of 1.72 cm s^− 1^, which is greater than that previously reported for the velocities of fungi (0.80 cm s^− 1^) [17] and plants (0.40 cm s^− 1^) [43] (Table 2). The large sedimentation velocity was associated with the large aerodynamic diameter of bacteria (Table 2). As discussed above, large bacterial aerodynamic diameters are likely attributed to their aggregation and/or attachment to other particles [14–16]. The different mechanisms of atmospheric liberation might explain why bacterial particles were larger than those of fungi and plants that release reproductive particles (e.g., spores and pollen grains) directly from their reproductive organs (e.g., sporangia and stamens) [80]. Due to their direct dispersal from reproductive organs, plant and fungal particles are less likely to be attached to other particles. Meanwhile, bacterial dispersal relies exclusively on external vectors, such as aeolian dust [15, 81, 82]. Indeed, we observed that dry deposition of bacteria was correlated well with wind velocities, although the results are not statistically significant, with Spearman’s *ρ* = 0.67–0.68 (Table S4), indicating that bacteria sedimented from the atmosphere are likely of aeolian origin. Thus, bacterial cells are more likely to be attached to other particles that might have higher material densities, e.g., > 2.0 g cm^− 3^ for abiotic particles [83], than the densities of biological materials themselves, e.g., 1.1–1.2 g cm^− 3^ for bacteria [84], 0.8–1.4 g cm^− 3^ for fungal spores [85], and 0.4–1.2 g cm^− 3^ for pollen grains [85]. Thus, bacterium-specific attachment to abiotic particles might explain the cause of their larger sedimentation velocities than those of fungi and plants.

### Precipitation dominates bacterial deposition from the atmosphere

On average, 95% of bacterial deposition is carried out via precipitation, indicating that precipitation is the dominant mode of atmospheric bacterial deposition. However, an anomalous tendency was observed in May during which only 4.1% of bacteria were precipitated and the remaining 95.9% were sedimented (Table S2). The large bacterial sedimentation in May is likely to be due to the small precipitation amount (Fig. 1b), in conjunction with the high atmospheric bacterial concentration (Fig. 3). The high bacterial concentration in May is possibly due to high concentrations of non-bacterial particles, such as Asian dust and airborne pollen, which are known to increase in Korea during the spring months [43, 86–88] and serve as external vectors for bacteria dispersal [15, 89–91].

### Distinct bacterial microbiotas between precipitation and sedimentation

We observed significantly different bacterial communities in precipitation from those in sedimentation and the air (Fig. 3c,d), while their memberships in sedimentation were not significantly different from those in the air (Fig. 3c). These findings are in congruence with a previous report that bacterial compositions in the free troposphere were similar to those in sedimentation (dry deposition) but not similar to those in precipitation (wet deposition) at high-elevation sites in Spain [39].

The bacterial genera specifically enriched in precipitation included *Aquabacterium*, *Bradyrhizobium*, *Burkholderia*, *Mucilaginibacter*, and *Novosphingobium*, while those abundant in both precipitation and sedimentation included *Massilia*, *Methylobacterium*, *Noviherbaspirillum*, and *Sphingomonas* (Fig. 2). *Noviherbaspirillum* and *Massilia* have been reported to be abundant in rainwater collected at high-elevation sites in Spain [39]. *Burkholderia*, *Massilia, Methylobacterium*, and *Mucilaginibacter* have been reported to be abundant in rainwater collected at mountain sites in China [36], while *Aquabacterium*, *Massilia*, *Methylobacterium*, *Novosphingobium*, and *Sphingomonas* are similarly abundant in cloud water collected at another mountain site in China [92]. Genera such as *Bradyrhizobium*, *Methylobacterium*, and *Sphingomonas* are also reported to be abundant in Arctic snow [31].

Nucleation activities are known for some of the bacterial genera abundantly detected in our precipitation samples. For instance, *Massilia* is known to have an ice-nucleation active strain [93]. The genus *Sphingomonas*, which contains several species previously classified in the genus *Pseudomonas*, is also known to have cloud condensation nuclei and ice-nucleation active strains [29, 94]. *Pseudomonas* was not abundant in our precipitation samples collected from spring to fall in Korea, which is in agreement with a previous study that analyzed rainwater collected during summer months in China [36]. Meanwhile, *Pseudomonas* is reported to be abundant in cloud water collected at a mountain site in France [30]. We do not know the exact causes of these inter-study variabilities. However, we expect that these variabilities might be due in part to spatiotemporal variabilities in the involvement of ice-nucleation active bacteria in processes of cloud formation. For instance, a larger involvement of ice-nucleation active bacteria is expected in colder continental climate zones where the cold rain process dominates, while smaller involvement is expected in warmer maritime climate zones where the warm rain process dominates.

### Do bacteria grow in the air?

We observed taxon-specific bacterial enrichment in precipitation (Fig. 2). As discussed, they might specifically be involved in and play a role in precipitation, for example, by serving as cloud nuclei, since some of the bacteria observed are known to have nucleation potential [29, 93, 94]. In addition, they might also reproduce and increase their populations in cloud droplets [95–100]. Though the in situ evidence is scarce, this hypothesis is supported by laboratory-based experiments that have demonstrated bacterial reproduction in droplets [99, 100]. Furthermore, the literature suggests that only selected microbes can thrive in clouds, and they are likely oligotrophic, psychrotolerant species that can utilize atmospheric components such as organic acids and alcohols as their nutrient sources [95, 98], with examples of such bacteria including *Burkholderiales*, *Methylobacterium*, and *Sphingomonas* [95]. We observed enrichment of *Burkholderia* in our precipitation samples (Fig. 2). Notably, *Burkholderia* were relatively scarce in the air (Fig. 4), indicating a possibility that they might selectively reproduce and increase in population in cloud droplets before precipitating from the atmosphere.

### Caveats

As discussed, bacteria might reproduce in cloud water. This means that bacteria might also reproduce in collected rainwater. We do not know exactly to what extent bacterial growth after sample collection has affected our calculations of wet deposition flux densities, even though we recovered collected rainwater samples immediately after each precipitation event and stored them at − 20 °C to minimize their growth. However, we expect that this artefact is small enough to capture the seasonal tendency of bacterial wet deposition, since a similar seasonal tendency was observed for amounts of precipitation (Fig. 1b), with a strong correlation observed (Fig. S2). It should be also noted that our finding of distinct microbiotas between dry and wet deposition (Fig. 5c,d) was unaffected by possible bacterial growth after sample collection, since the differences were confirmed not only in bacterial structures but also in their memberships, which are thought to be independent of their growth. In addition, bacteria might also reproduce and/or die on substrates of air and dry deposition samplers during a 1-month sampling period. However, we expect that the bacterial growth was small enough due to harsh conditions, e.g., desiccation caused by air flow created by the sampler. For instance, a study [101] has reported the monotonical increase of bacterial DNA and stable community structures on an air filter throughout a 21-week sampling period, indicating the stability of bacterial DNA on air filters and substrates at least for a duration of several weeks.

## Conclusions

This study investigated atmospheric bacterial deposition. We found that bacteria had large aerodynamic diameters, likely due to the aggregation and/or attachment to other larger particles, which likely results in their observed large sedimentation velocities from the atmosphere. On average, precipitation contributed to 95% of total bacterial deposition in our sampling site in Korea. The large contribution of precipitation might be in part due to their large-size particles, which can serve as efficient nuclei in clouds (e.g., as giant cloud condensation nuclei). Moreover, we observed distinctly different microbiotas between precipitation and sedimentation, which might indicate specific bacterial involvement and/or selective bacterial growth in clouds. Overall, our findings provide a novel insight into how bacteria participate in atmospheric processes and the global cycling of substances on Earth, including the water cycle.

## Methods

### Precipitation, sedimentation, and air samples

Previously collected samples on a rooftop (about 20 m above ground level) of a building in Seoul in Korea (37°27′55.0″N; 126°57′17.7″E) from May to November 2015 [17, 43] were analyzed here. Briefly, the sampling site was on a university campus in a hilly suburban forested area in the Seoul Special City, the capital megacity of South Korea. Detailed information of our sampling site, such as the vegetation, topography, climate characteristics, and weather conditions such as temperature, relative humidity, wind velocity, and precipitation, is available in our previous publications [17, 43].

Briefly, deposition samples were collected using an automatic dry and wet deposition sampler [44]. The sampling heights of dry and wet deposition samplers were 1.1 and 0.9 m from the raised floor which is about 2.3 m above the rooftop of the building, respectively. Each sedimentation sample was collected on a quartz fiber substrate (47 mm diameter, QR-100; Advantec Tokyo Kaisha, Ltd., Tokyo, Japan) placed on a knife-edge surrogate surface [102] for a duration of 1 month, while each precipitation sample was collected through a polypropylene funnel into a 1 L polypropylene bottle, from which collected rainwater was recovered into 250 mL polypropylene bottle(s) immediately after each precipitation event, which typically continued for one to several days. The total collected amounts of precipitation were 620, 1520, 3110, 1400, 440, 770, and 1870 mL for May, June, July, August, September, October, and November, respectively, which correspond to precipitation flux densities of 3.7, 9.1, 18.6, 8.4, 2.6, 4.6, and 11.2 mL cm^− 2^ month^− 1^, respectively (Fig. 1b). Duplicates and travel blanks were also collected. For each sedimentation travel blank, a clean quartz fiber substrate was loaded onto the dry deposition sampler, immediately unloaded, and then placed into a sterile Petri dish and transported to the laboratory. For each precipitation travel blank, 50 mL of ultrapure water was poured into the polypropylene funnel of the wet deposition sampler, then immediately recovered from the 1 L polypropylene bottle and decanted into a sterile 50 mL tube and transported to the laboratory. The collected sedimentation and precipitation samples, including duplicates and travel blanks, were stored at − 20 °C until subsequent sample processing.

Air samples were collected using an eight-stage Andersen sampler (AN-200; Sibata Scientific Technology Ltd., Tokyo, Japan) with an air flow rate adjusted at 28.3 L min^− 1^, in which atmospheric particles were size-fractionated and collected on glass fiber substrates (80 mm diameter, QR-100; Advantec Tokyo Kaisha, Ltd., Tokyo, Japan) for size bins of *d*_a_ = 0.43–0.65, 0.65–1.1, 1.1–2.1, 2.1–3.3, 3.3–4.7, 4.7–7.0, 7.0–11, and > 11 μm. The sampling height was about 0.4 m above the rooftop of the building. Air sampling in August failed due to an intense precipitation event. Due to the small bacterial biomass, substrates loaded on the stages of *d*_a_ = 0.43–0.65, 0.65–1.1, and 1.1–2.1 μm were not analyzed. The sampled substrates were kept at − 20 °C.

### DNA extraction

For sedimentation samples, including duplicates and travel blanks, one quarter area of each sampled substrate was used for DNA extraction. For precipitation samples, including duplicates, rainwater samples collected at different rainfall events within a same 1-month sampling period were combined and filtered to recover particulate matters, including bacterial cells, onto a same membrane filter attached to a sterile MiroFunnelTM Filter Funnel (47 mm diameter, 0.45 μm pore size, GN-6 Mericel® white gridded membrane; Pall Corporation, NY, USA). Similarly, each 50 mL precipitation travel blank was filtrated through a membrane filter. One quarter area of each filter was used for DNA extraction. For air samples, one eighth area of each sampled substrate was used for DNA extraction. The DNA was extracted using a PowerMax® Soil DNA Isolation Kit (Mobio Laboratory, CA, USA) with a modified physical disruption step with supplementary 0.1 mm diameter glass beads (300 mg) and 0.5 mm diameter glass beads (100 mg) [103] by a bead beater (BioSpec Products, OK, USA). Then, the DNA was purified and eluted into 50 μL of TE (10 mM Tris-HCl, 1 mM EDTA, pH = 8.0) according to the kit protocol.

### Quantitative PCR

Universal bacterial qPCR assays were performed with forward primer 5′- TCCTACGGGAGGCAGCAGT-3′, reverse primer 5′-GGACTACCAGGGTATCTAATCCTGTT-3′, and a TaqMan probe (6-FAM)-5′-CGTATTACCGCG GCTGCTGGCAC-3′-(BHQ1), targeting 331 to 797 region of *Escherichia coli*’s 16S rRNA gene [104]. Each of the 50 μL qPCR mixtures contained 25 μL of 2× TaqMan Universal PCR Master Mix (Life Technologies, CA, USA), 1 μL of the 10 μM probe, 1 μL of each of the 10 μM primers, 20 μL of nuclease-free water, and 2 μL of a DNA template. PCR assays were performed on an QuantStudio™ 6 Flex Real-time PCR system (Applied Biosystems, Waltham, MA, USA) with a condition of 12 min at 95 °C for initial denaturation, followed by 45 cycles of 15 s at 95 °C for denaturation, 45 s at 56 °C for annealing, and 90 s at 72 °C for extension. A standard template was prepared by amplifying the DNA extract from the cultured *E. coli* strain (ATCC 25922) by conventional PCR, using the same primers for qPCR. The amplicon was purified and quantified for its mass concentration by a Quant-iT PicoGreen dsDNA reagent kit (Life Technologies), from which the number concentration of the amplified 16S rRNA gene fragments was calculated based on their length (i.e., 466 bp). The amplicon was serially diluted to prepare standard templates with concentrations ranging from 10^1^ to 10^6^ fragment copy number (CN) μL^− 1^. All assays were technically in triplicate. No inhibition was found according to a method reported elsewhere [103]. Here, a 10% DNA extraction efficiency was assumed to calculate the bacterial quantities on the sampled substrates or filters [103]. The results were reproducible with an 81% cumulative coefficient of variation based on duplicates for deposition samples (Fig. S2a).

### DNA sequencing

DNA libraries were prepared according to a method reported elsewhere [105]. Briefly, the same primer sets, which were used for qPCR, with adaptor sequences for Illumina MiSeq, were used to amplify bacterial 16S rRNA genes. Each reaction mixture (30 μL) contained 15 μL of the 2× PCR Solution Premix Taq™ (Takara Bio Inc., Otsu, Shiga, Japan), 1 μL of each of the 10 μM primers, and 1 μL of a DNA template. PCR was performed with a condition of 5 min at 95 °C for initial denaturation, followed by 35 cycles of 15 s at 95 °C for denaturation, 45 s at 56 °C for annealing, and 90 s at 72 °C for extension, and completed by extension for 10 min at 72 °C. No travel blanks were amplified. The amplicons were purified by AMPure XP beads (Beckman Coulter, Inc., CA, USA). The purified amplicons were indexed using the Nextera XT Index kit (Illumina, Inc., CA, USA) with a condition of 3 min at 95 °C for initial denaturation, followed by 8 cycles of 30 s at 95 °C for denaturation, 30 s at 55 °C for annealing, and 30 s at 72 °C for extension, and completed by extension for 5 min at 72 °C. The resultant amplicons were purified by AMPure XP beads and quantified using the Quant-iT PicoGreen dsDNA reagent kit (Life Technologies). The quantified amplicons were normalized, pooled with an internal control PhiX (30%), and loaded onto a v3 600 cycle-kit reagent cartridge (Illumina) for 2 × 300 bp paired-end sequencing by Illumina MiSeq.

### DNA sequence processing and analyses

MiSeq Reporter version 2.5 (Illumina) was used to trim the primer and multiplexing barcode sequences and remove reads with quality scores below 20. Next, USEARCH version 11.0.667 [106] was used to join the forward and reverse reads and then remove low-quality reads with > 1.0 expected errors and/or those with lengths less than 200 bp. After quality trimming, the UPARSE-OTU algorithm [107] was used to cluster unique sequences into 97% operational taxonomic units (OTUs) and remove chimeric reads. Using the SINTAX algorithm [108], each OTU was taxonomically assigned against RDP training set v16 (rdp_16s_v16.fa.gz) [109] with a cutoff confidence value of 0.5 [110]. For diversity analyses, libraries were rarefied to 8000 sequence reads using the “phyloseq” package [111] on R version 3.4.0, which was also used to calculate α-diversity measures and distance matrices for β-diversity analyses. The Jaccard indices for assemblage memberships and Bray–Curtis similarity coefficients for assemblage structures were calculated, for which parsimony tests (P-tests) were performed to compare the bacterial assemblages between air, dry, and wet deposition samples using mothur version 1.39.5 [77]. The reproducibility of characterizing bacterial assemblages was confirmed based on duplicates for deposition samples (Fig. S3b,c), with smaller intra-sample distances than inter-sample distances in terms of both their memberships and structures (Fig. S3d,e).

### Data analyses

Taxon-specific bacterial concentrations were obtained by the multiplication of sequence relative abundances by the total bacterial concentrations obtained by qPCR [66, 112]. The geometric mean of the aerodynamic diameter of a given particle size distribution was computed using the GM calculator, version 1.0 [66]. Then, the arithmetic mean of the computed monthly geometric mean aerodynamic diameter was calculated to provide an aerodynamic diameter representative for each bacterial taxon. The bacterial sedimentation velocity (*V*_d_) (cm s^− 1^) was calculated according to the following equation:

1 $$ {V}_d=\sum \limits_{j=1}^6{F}_j/\sum \limits_{j=1}^6\sum \limits_{i=1}^5{N}_{j,i} $$

where *F*_*j*_ is the sedimentation flux density (CN cm^− 2^ month^− 1^) quantitated for the *j*th month by the dry deposition sampler, and *N*_*j,i*_ is the volumetric concentration (CN m^− 3^) in the *i*th particle size bin for the *j*th month measured by the Andersen sampler. The August data were excluded due to the failure of air sampling. For this calculation, the units were converted from m to cm, and from month to second if necessary. The calculated sedimentation velocities were compared with theoretical terminal settling velocities (*V*_Stk_), given by Stokes’ law:

2 $$ {V}_{\mathrm{Stk}}={\rho}_0{d}_a^2g/18\eta $$

where *ρ*_0_ is the standard density (1.0 g cm^− 3^), *g* is the acceleration of gravity (980 cm s^− 2^), and *η* is the viscosity of air (1.8 × 10^− 5^ Pa s).

## Supplementary Information

**Additional file 1: Table S1.** Numbers of high-quality sequence reads by Illumina MiSeq. **Table S2.** Flux densities (CN cm-2month-1) of atmospheric bacterial deposition in dry and wet forms. The percentage values in parentheses indicate relative contributions. **Table S3.** Correlation coefficients between peak aerodynamic diameters of total bacterial particles and parameters related to weather conditions observed in each sampling month. **Table S4.** Correlation coefficients between deposition flux densities and airborne concentrations of bacteria and parameters related to weather conditions. The Spearman’s rank correlation coefficients are shown with or without asterisk (*) representing statistical significance (*p*< 0.05). **Figure S1.** Rarefaction curves based on 97% OTUs of 16S rRNA gene sequences. a Deposition samples. b Air samples. **Figure S2.** Relationship between amounts of precipitation and flux densities of wet deposition of total bacteria. **Figure S3.** Reproducibility based onduplicates of deposition samples.

## Data Availability

Raw sequence data are available under the BioProject number PRJNA603417 of NCBI.

## Abbreviations

OTUs: Operational taxonomic units
QPCR: Quantitative polymerase chain reaction
CN: Copy number
CFU: Colony forming unit
