# Supplementary material for: Falling bacterial communities from the atmosphere
Source: Environ Microbiome. 2020 Dec 10;15:22. doi: 10.1186/s40793-020-00369-4 (PMC8066439; doi:10.1186/s40793-020-00369-4)
Supplement: Supplementary file 1 — Additional file 1: Table S1. Numbers of high-quality sequence reads by Illumina MiSeq. Table S2. Flux densities (CN cm-2month-1) of atmospheric bacterial deposition in dry and wet forms. The percentage values in parentheses indicate relative contributions. Table S3. Correlation coefficients between peak aerodynamic diameters of total bacterial particles and parameters related to weather conditions observed in each sampling month. Table S4. Correlation coefficients between deposition flux densities and airborne concentrations of bacteria and parameters related to weather conditions. The Spearman’s rank correlation coefficients are shown with or without asterisk (*) representing statistical significance (p< 0.05). Figure S1. Rarefaction curves based on 97% OTUs of 16S rRNA gene sequences. a Deposition samples. b Air samples. Figure S2. Relationship between amounts of precipitation and flux densities of wet deposition of total bacteria. Figure S3. Reproducibility based onduplicates of deposition samples. [file 40793_2020_369_MOESM1_ESM.pdf]

Supporting Information for:

Falling bacterial communities from the atmosphere

Submitted to:

Environmental Microbiome

Cheolwoon Woo[1] and Naomichi Yamamoto[1,2*]

1. Department of Environmental Health Sciences, Graduate School of Public Health, Seoul National University, Seoul 08826, Republic of Korea
2. Institute of Health and Environment, Seoul National University, Seoul 08826, Republic of Korea

* Correspondence. nyamamoto@snu.ac.kr

Table S1. Numbers of high-quality sequence reads by Illumina MiSeq.

Sample ID	Type	Start date (yymmdd)	End date (yymmdd)	Sample type	Duplicate	Number of reads
5A0	Air	150501	150531	$d_a > 11$ μm	-	52248
5A1				$d_a = 7–11$ μm	-	37286
5A2				$d_a = 4.7–7$ μm	-	45772
5A3				$d_a = 3.3–4.7$ μm	-	38347
5A4				$d_a = 2.1–3.3$ μm	-	46148
6A0		150601	150630	$d_a > 11$ μm	-	41529
6A1				$d_a = 7–11$ μm	-	53060
6A2				$d_a = 4.7–7$ μm	-	54831
6A3				$d_a = 3.3–4.7$ μm	-	40052
6A4				$d_a = 2.1–3.3$ μm	-	42722
7A0		150701	150731	$d_a > 11$ μm	-	41597
7A1				$d_a = 7–11$ μm	-	51937
7A2				$d_a = 4.7–7$ μm	-	40134
7A3				$d_a = 3.3–4.7$ μm	-	41281
7A4				$d_a = 2.1–3.3$ μm	-	40761
9A0		150901	150930	$d_a > 11$ μm	-	40280
9A1				$d_a = 7–11$ μm	-	46119
9A2				$d_a = 4.7–7$ μm	-	31585
9A3				$d_a = 3.3–4.7$ μm	-	8425
9A4				$d_a = 2.1–3.3$ μm	-	33284
10A0		151001	151031	$d_a > 11$ μm	-	48858
10A1				$d_a = 7–11$ μm	-	41645
10A2				$d_a = 4.7–7$ μm	-	55277
10A3				$d_a = 3.3–4.7$ μm	-	15502
10A4				$d_a = 2.1–3.3$ μm	-	46877
11A0		151101	151130	$d_a > 11$ μm	-	47856
11A1				$d_a = 7–11$ μm	-	47465
11A2				$d_a = 4.7–7$ μm	-	52092
11A3				$d_a = 3.3–4.7$ μm	-	49909
11A4				$d_a = 2.1–3.3$ μm	-	29987
5D1	Deposition	150501	150531	dry	1	38806
5D2				dry	2	41967
5W1				wet	1	22981
5W2				wet	2	38081
6D1		150601	150630	dry	1	39277
6D2				dry	2	41960
6W1				wet	1	36165
6W2				wet	2	37879
7D1		150701	150731	dry	1	42011
7D2				dry	2	45269
7W1				wet	1	34810
7W2				wet	2	37300
8D1		150801	150831	dry	1	61239
8D2				dry	2	42128
8W1				wet	1	33972
8W2				wet	2	30064
9D1		150901	150930	dry	1	29606
9D2				dry	2	44971
9W1				wet	1	31381
9W2				wet	2	40208
10D1		151001	151031	dry	1	45415
10D2				dry	2	41776
10W1				wet	1	59654
10W2				wet	2	38263
11D1		151101	151130	dry	1	40632
11D2				dry	2	53050
11W1				wet	1	32234
11W2				wet	2	38332

Table S2. Flux densities (CN cm^{-2} $month^{-1}$) of atmospheric bacterial deposition in dry and wet forms. The percentage values in parentheses indicate relative contributions.

Month	Dry	Wet
May	6513662 (95.9%)	277179 (4.1%)
June	26287 (0.1%)	23044749 (99.9%)
July	342640 (0.4%)	77112468 (99.6%)
August	29160 (0.7%)	4136328 (99.3%)
September	189826 (25.0%)	568420 (75.0%)
October	144493 (12.6%)	1004207 (87.4%)
November	219554 (0.7%)	29889532 (99.3%)

Table S3. Correlation coefficients between peak aerodynamic diameters of total bacterial particles and parameters related to weather conditions observed in each sampling month.

Weather parameters [a]	Spearman's rank correlation coefficient [b]
Highest temperature	0.03
Mean temperature	0.09
Lowest temperature	0.09
Highest RH	–0.14
Mean RH	–0.60
Lowest RH	–0.77
Highest wind velocity	–0.32
Mean wind velocity	–0.23
Precipitation	–0.54

[a] The weather parameters reported by our previous study [1] are used. The highest and lowest temperatures, relative humidity (RH) and wind velocities are based on 1-min monitoring interval.

[b] All of the results are not statistically significant ($p > 0.05$).

Table S4. Correlation coefficients between deposition flux densities and airborne concentrations of bacteria and parameters related to weather conditions. The Spearman's rank correlation coefficients are shown with or without asterisk (*) representing statistical significance (p < 0.05).

Weather parameters [a]	Dry deposition	Wet deposition	Total deposition	Airborne bacteria
Highest temperature	–0.11	0.32	0.39	0.89*
Mean temperature	–0.32	0.21	0.07	0.71
Lowest temperature	–0.32	0.21	0.07	0.71
Highest RH	0.32	0.71	0.93*	0.03
Mean RH	–0.04	0.86*	0.57	–0.26
Lowest RH	–0.14	0.61	0.21	–0.09
Highest wind velocity	0.68	0.14	0.63	0.29
Mean wind velocity	0.67	0.23	0.77*	0.58
Precipitation	0	0.96*	0.89*	0.26

[a] The weather parameters reported by our previous study [1] are used. The highest and lowest temperatures, relative humidity (RH) and wind velocities are based on 1-min monitoring interval.

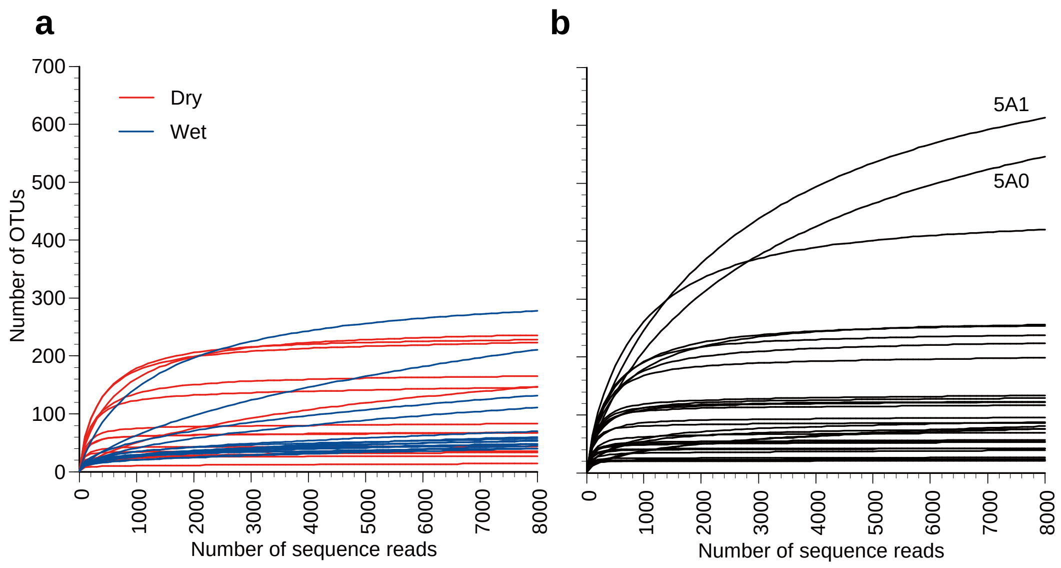

Figure S1. Rarefaction curves based on 97% OTUs of 16S rRNA gene sequences. **a** Deposition samples. **b** Air samples.

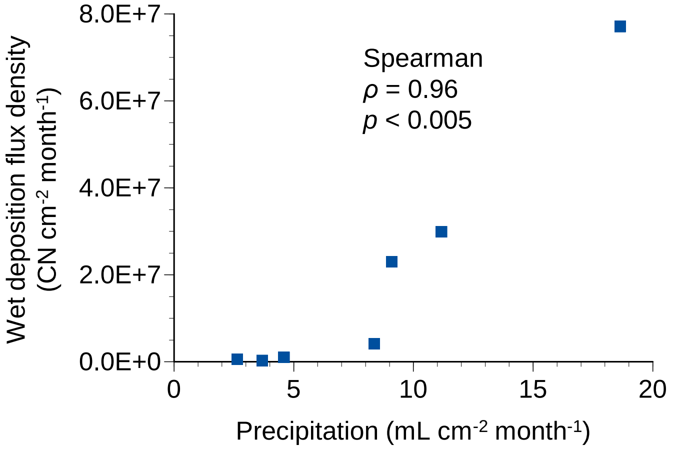

Figure S2. Relationship between amounts of precipitation and flux densities of wet deposition of total bacteria.

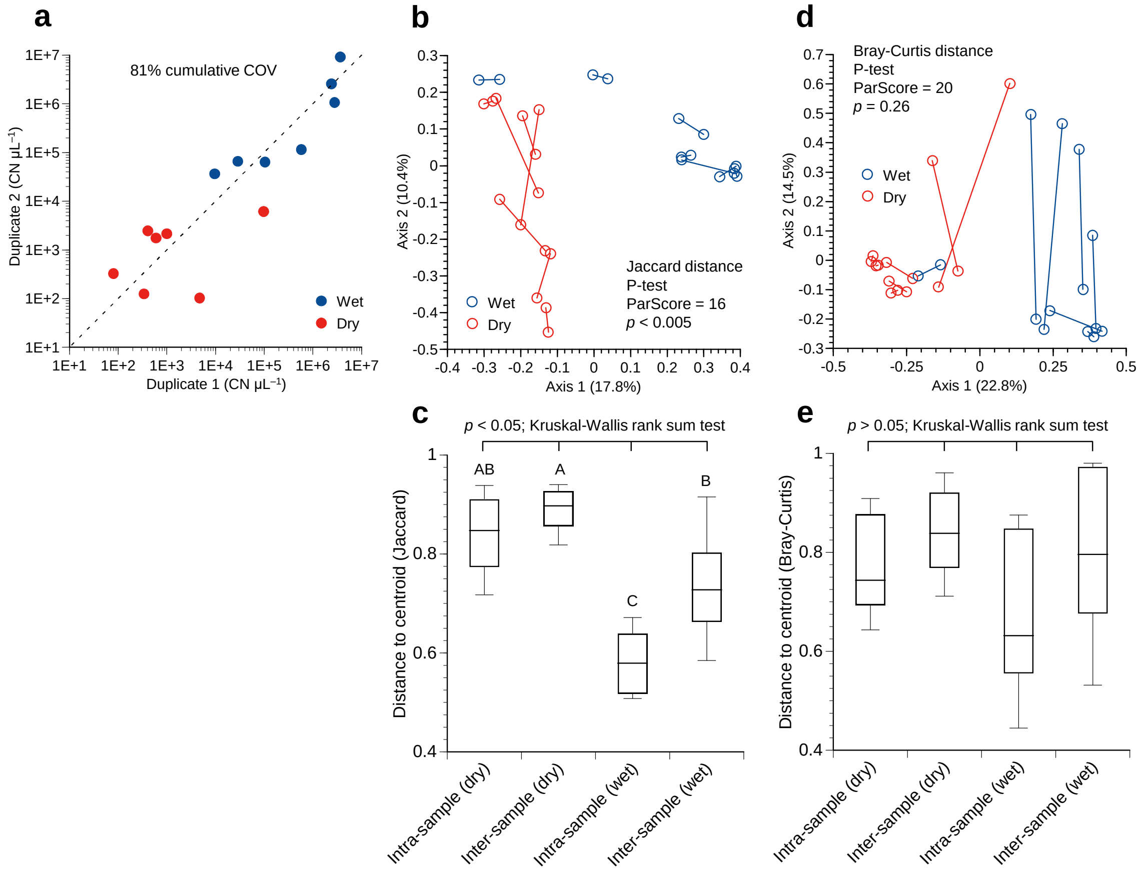

Figure S3. Reproducibility based on duplicates of deposition samples.
a Number of bacterial 16S rRNA gene copies in 1 μL of each DNA extract as quantitated by qPCR. The dashed line indicates 1:1. The results were reproducible with 81% cumulative coefficient of variation (COV) based on an arithmetic scale. **b** Principal coordinate analysis plot of bacterial memberships in terms of Jaccard indices based on 97% OTUs. Duplicates of each sample are connected by a line. **c** Boxplot showing distributions of pair-wise Jaccard distances across libraries. Different letters (i.e., A, B, and C) indicate significant differences based on the *post hoc* pair-wise Wilcoxon rank-sum tests with Bonferroni correction. **d** Principal coordinate analysis plot of bacterial structures in terms of Bray-Curtis distances based on 97% OTUs. Duplicates of each sample are connected by a line. **e** Boxplot showing distributions of pair-wise Bray-Curtis distances across libraries.

Reference

1. Woo C, An C, Xu S, Yi S-M, Yamamoto N. Taxonomic diversity of fungi deposited from the atmosphere. ISME J. 2018;12(8):2051–2060.
